# Evaluation of Anterior-Posterior Spine Curvatures and Incidence of Sagittal Defects in Children and Adolescents Practicing Traditional Karate

**DOI:** 10.1155/2019/9868473

**Published:** 2019-04-28

**Authors:** Walicka-Cupryś Katarzyna, Szeliga Ewa, Guzik Agnieszka, Mrozkowiak Mirosław, Niewczas Marta, Ostrowski Paweł, Tabaczek-Bejster Iwona

**Affiliations:** ^1^Institute of Physiotherapy, Faculty of Medicine, University of Rzeszow, Warszawska 26A Street, 35-205 Rzeszow, Poland; ^2^Faculty of Physical Education, Health and Tourism, Kazimierz Wielki University in Bydgoszcz, Chodkiewicza 30 Street, Bydgoszcz, Poland; ^3^Faculty of Physical Education, Department of Sport, University of Rzeszow, Towarnickiego 3 Street Rzeszow, Poland; ^4^Department of Obstetrics and Emergency Medicine, Faculty of Medicine, University of Rzeszow, Pigonia 6 Street, 35-310 Rzeszów, Poland

## Abstract

**Background and Study Aim:**

To evaluate anterior-posterior spine curvatures and incidence of sagittal defects in children and adolescents practicing traditional Karate.

**Material and Methods:**

152 people aged 6-16 yrs, mean age 10.5±3.03. The study group (SG), subjects attending traditional Karate classes for min one year, 60 minutes twice a week (76 people). The control group (CG) randomly selected on 1:1 basis to match SG. The anterior-posterior curvatures were measured with a gravitational inclinometer. Sauder's norms were used to assess postural defects. Body mass, height, and BMI were measured. Mann-Whitney U-test/Student's t-test for independent variables, *χ*^2^ test, and Cramer V test were used in statistical analysis.

**Results:**

Significant differences were observed between the groups in lumbosacral inclination ALPHA1 p<0.001; karateka had significantly lower ALPHA 1 and greater thoracic inclination (GAMMA TH/L) p=0.23. In study group, lumbosacral angle flattening (ALPHA 2) (81.6%) was greater than in CG (56.6%). ALPHA 2 (43.4%) was more frequently within the norm in CG. Increased ALPHA 2 was reported only among SG (2.6%). Differences were statistically significant (*χ*^2^ (2) = 15.23 p<0.001) and of moderate power (Cramer V=0.31). Regarding thoracic kyphosis and lumbar lordosis, there were no statistically significant differences between the groups.

**Conclusions:**

Traditional Karate affects pelvic tilt leading to posterior tilt; it correlates with somatic parameters: height, mass, and BMI in terms of spine curvatures. The size of the lumbar lordosis and thoracic kyphosis in karatekas is comparable to that of those not practicing sport. Frequent incidence of reduced pelvic tilt in karatekas requires implementing exercises activating anterior tilt during training session.

## 1. Introduction

Proper body posture is very important for human health. It is influenced by various factors that can be divided into morphological, psychological, and environmental ones. Daily habits, lifestyle, as well as the nature of physical activity, including various sports fields are also important [1–3]. Athlete's body posture analysis is the subject of numerous studies [4–9]. However, this topic in most cases focuses on the adverse, traumatic impact of many sports [10–13]. However, the influence of martial arts, including karate, on the spine curvatures in children is described only in a few reports [14].

Karate is one of the most popular martial arts in the world, practiced by children, adolescents, adults, and seniors [15–17]. Karate originated in Japan and literally means “empty hand” as it was created as a method of self-defence without weapons. Since its creation, this art has developed considerably. Some styles have retained the aspect of fight; others have changed the character into the utilitarian system of fight intended for psychophysical improvement, where the spiritual development achieved by the demanding physical exercise is crucial [14, 15, 18]. Karate includes three basic techniques: throws, blows, and blocks (offensive and defensive), as well as three basic directions: traditional karate, karate as a sport with self-defence, and karate as a method of psychophysical improvement [15, 19]. High-level technical skills are necessary, and to make the right moves in attack and defence, high precision and speed are required. In addition, it is a sport which by improving cognitive abilities and learning processes affects the organization of motor behaviour, while providing a high degree of coordination and a quick and correct response to visual stimuli [19–23].

Karate is a method of complex movements, where the techniques of attack and defence are characterized by maximum intensity, intertwined with short breaks, which makes the character of this sport comparable to intermittent and intensive exercises [15]. Therefore, the question arose: Can performing this kind of physical activity with a specific volume and intensity by children and teenagers affect the posture of the body? The answer to the aforementioned question has become the motivation to undertake this research. The review of the literature of the subject shows that this influence is unfavourable in the case of sports that are performed for a longer period of time and of considerable intensity. These reports emphasize the importance of sports with asymmetric muscle work, and this group includes martial arts [24]. Other works on the assessment of body posture among children aged 7 up to 10 years practicing karate showed a significant increase in thoracic kyphosis and lumbar lordosis [14]. A publication was found reporting the positive impact of traditional karate on postural stability [18]. The aim of the study is to evaluate the anterior-posterior spine curvatures and the incidence of sagittal defects in children who practice traditional karate and in children not practising any sport.

## 2. Material and Methods

### 2.1. Participants

152 people aged 6 to 16 years, mean age 10.5 ± 3.03, were enrolled in the study. The study group included people attending traditional karate classes for at least one year, twice a week for 60 minutes (76 people, 120 boys and 32 girls). The study was carried out prior to a traditional karate class in Rzeszów in 2015-2017. The control group were children not physically active according to the MVPA (Moderate-to-Vigorous Physical Activity) index [25], who at the same time did not regularly do any sport. The control group was selected on 1:1 basis, in terms of age and gender, from the area of south-eastern Poland assessed in 2015-2017. Written informed consent was obtained from all the parents or legal carers of the children, after being informed of the study objectives. Participants aged 16 years signed an informed consent form as well. The study was approved by the Medical Faculty Bioethics Commission, at the University of Rzeszów. Experimental conditions conformed to the Declaration of Helsinki.

The inclusion criteria in the study group were the consent of parents and children for examination, regular participation in traditional karate classes for more than 1 year, and lack of neurological and orthopaedic diseases affecting the shape of curvatures of the spine. Inclusion criteria of in the control group were as follows: age, gender matched to the study group, lack of neurological and orthopaedic diseases affecting the posture, and lack of sufficient level of physical activity with MVPA. Moderate physical activity was defined as more than 1 hour a day of cumulative motor activity in the 5 days of the week, whereby motor activity is understood as all forms of physical activity that speed up respiration, e.g., PE classes, sports activities, intense activity during school breaks, walking, and running to or from school. It has been assumed that the response of 5 days or more (MVPA ≤ 5) means physical activity satisfying the minimum needs of adolescents [25, 26]. At the same time, children from the control group did not do any sport more than twice a week.

Exclusion criteria from both groups were the lack of consent, lower limb injuries (dislocation, sprain) one month before the examination, fractures of the lower limbs in the last 6 months, tactile hypersensitivity resulting in inability of adopting free, habitual posture, and simultaneous regular practice of a sport other than traditional karate.

The flow of the subjects were as follows: Based on the eligibility criteria, 102 karatekas were preliminarily qualified for the study. However, 17 people did not report to the study, 3 people refused to take part on the examination day, and 5 showed tactile hypersensitivity preventing examination. Finally, 76 people from the study group participated in the study.

### 2.2. Anthropometric Measurements

All the measurements were performed on the same day, starting with anthropometric measurements. Body height was measured with Seca 213 mobile stadiometer, with an accuracy of 0.1 cm. Body mass was measured using electronic scale OMRON BF 500, with an accuracy of 0.1 kg. The measurements were performed in standard conditions; children in underwear and barefoot were standing in upright position, without bending knees. Anthropometric measures of both groups are shown in Table 1.

### 2.3. Body Posture Measurements

Body posture was examined using inclinometer techniques which is reliable [27], handy, and affordable; therefore, it is frequently used in examinations [28–30]. The research methodology was performed in accordance with the guidelines in five topographic points [31]. Following the palpation of the points (spinous processes, transition of kyphosis into lordosis, and posterior iliac spines), they were marked with a dermatograph, the procedure being consistent with other similar studies [28, 32, 33].

The assessment was made in a free standing position, with legs extended in the knees with the eyes directed to the point placed straight ahead of the subject at a distance of 1.5 m. The following instructions were given: “stand in a comfortable manner”; “do not bend your knees”; and “look straight.” The children were not instructed to straighten up [29, 30]. When the subject corrected their body posture, the measurement was repeated so that functional defects were also noted and these subjects were excluded from the analysis.

Correct measurement was when the inclinometer was targeted vertically and the measurement observation was perpendicular to the device. The tests were performed by a physiotherapist with five years of experience as an operator of the equipment and 15 years of experience in testing body posture.

**Figure 1:**
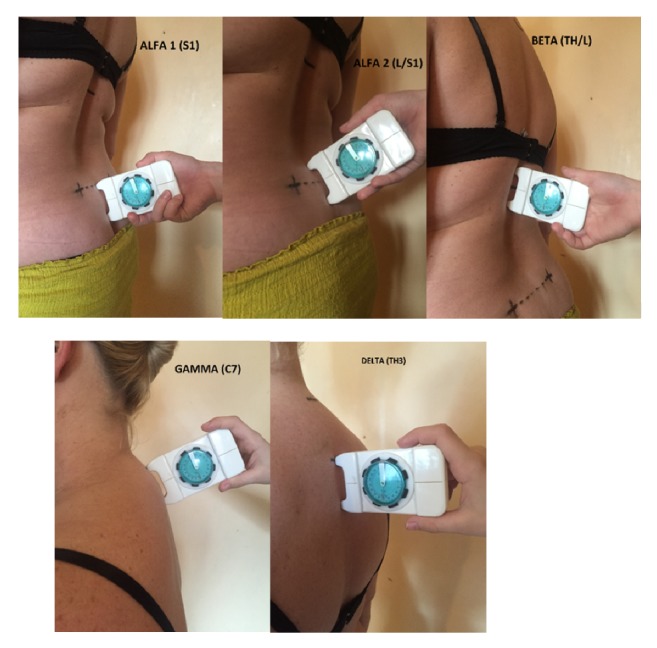
Measurement with gravity inclinometer.

The following parameters were examined by the study: ALPHA angle 1 (ALPHA 1), sacral inclination (the upper inclinometer foot in the middle of the intervertebral space on the line connecting of the posterior superior iliac spines), the ALPHA angle 2 (ALPHA 2), the medial point of the sacrum on the line connecting the so-called Venus dimples (between the inclinometer feet), the BETA angle (BETA Th/L) intervertebral space Th12- L1 (inclinometer on thoracolumbar transition in the middle between the inclinometer feet), GAMMA angle (GAMMA C7) intervertebral space C7-Th1 (top inclinometer foot on C7), DELTA angle (DELTA Th3) intervertebral space Th3-Th4, inclinometer in the plateau of kyphosis, the upper foot right at the end of the convexity forming Dowager's hump. Lumbar lordosis angle was calculated as (S2) + (Th/L), the thoracic kyphosis angle TKA, (Th/ L) + (C7); Dowager's hump angle (TKA DH), (C7) + (Th3) [Figure 1].

Changes in the curvature of the spine were evaluated on the basis of the general guidelines for inclinometers acc. to Sauder's, where the following values were assumed normal: lumbosacral angle (ALPHA angle 1) 15–30°; curvature of the lumbar lordosis, TKA (ALPHA angle 2 + BETA angel) 30–40°; curvature of the thoracic kyphosis (BETA angle + GAMMA angle) 30–40° [34, 35].

### 2.4. Statistics

Statistical analyses of the collected material were performed using Statistica 13.1 from StatSoft. Both parametric and nonparametric tests were applied in the analysis of the variables. The choice of parametric test depended on the fulfilment of its basic assumptions, i.e., conformity of the distributions of the examined variables with normal distribution, which was verified with Shapiro-Wilk W-test. The evaluation of the significance level of differences between groups was performed with Mann-Whitney U test or Student t-test for independent variables, *χ*^2^ test; Cramer's V was used to assess the incidence of spinal problems in the sagittal plane. Values of statistics for p < 0.05 were considered statistically significant, besides which the following principles were adopted to evaluate test probability: p < 0.001, the appearance of a really very high statistically significant dependence (*∗∗∗*); 0.001 V p <0.01, the appearance of a really high statistically significant dependence (*∗∗*); 0.01 V p < 0.05, the appearance of a statistically significant dependence (*∗*).

## 3. Results

Significant differences were observed between the groups in the inclination of lumbosacral section ALPHA1 p <0.001; karatekas had significantly lower ALPHA 1 inclination and greater inclination of the upper thoracic section (GAMMA TH / L) p=0.023 (Table 2).

In the case of the subjects from the karate group, ALPHA 2 flattening was more frequently reported (81.6%) than in the control group (56.6%). In the control group, normal ALPHA 2 was more frequently observed (43.4%). Increased ALPHA 2 was reported only among the karate group (2.6%). The differences were statistically significant (*χ*^2^ (2) = 15.23 p <0.001) and of moderate power (V Cramera = 0.31) (Table 3). In terms of curvature, thoracic kyphosis and lumbar lordosis, there were no statistically significant differences between the groups (Tables 4 and 5).

Analysing the influence of body mass and height and BMI on the parameters characterizing curvature of the spine, statistically significant relationships were demonstrated in the following range: ALPHA 1 angle, which increased with body weight and BMI in the study group; lumbosacral angle (ALPHA 2), increased with the body height in the study group; the angle of thoracic kyphosis between the scapulae (DELTA Th3) decreased with the increase of body weight and height in the study group. In the control group, there was a significant relationship between the inclination of the upper thoracic segment (GAMMA C7) and the increase in body weight and height and BMI (Table 6).

## 4. Discussion

Numerous reports on the impact of martial arts, including karate on the biomechanical, morphological, or psychophysical state of the body are available in the literature [21, 36–38]. On the other hand, results of research on the impact of regular karate training on the posture of children [14] are sporadic. This observation has become the motivation to undertake this research, which aims to characterize the anterior-posterior spine curvatures in children attending traditional karate classes for a minimum of a year.

Our studies showed statistically significant differences in the size of sacral angle parameter (ALPHA 1). The karatekas had significantly lower anterior tilt of sacrum and greater inclination of the thoracic-lumbar transition compared to the control group. In the case of the subjects from the karate group, sacral flattening was more frequently reported (81.6%) than in the control group (56.6%). Increased inclination of lumbosacral section was reported only among the karate group (2.6%). In terms of curvature, thoracic kyphosis and lumbar lordosis, there were no statistically significant differences between the groups.

Mucha et al. reported that unilateral training often leads to reduction in the physiological curvature of the spine, which is the result of excessive strengthening of the Erector spina muscles [24]. Analysing the case of the posture of a 21-year-old master-class athlete in ju-jitsu training martial arts for 10 years, the authors showed that the subject had deepened lumbar lordosis (51°) and a slightly deviated spine from the vertical line. At the same time, they indicate that these are small deviations from the physiological state and can be considered typical for athletes practising martial arts [24]. However, the above results relate to the subject being tested in adolescence and not in childhood. The only research currently available in the literature on the impact of martial arts on the body posture in children was conducted by Drzał-Grabiec and Truszczyńska. The authors showed that children practicing Kyokushin karate for over two years are characterized by a significant deepening of physiological thoracic kyphosis and lumbar lordosis, which was not found in our study of children practicing traditional karate. They also reported that the body posture of children practicing karate was characterized by a greater angle of the thoracic-lumbar region, which, in comparison to the control group, is consistent with our research. 50 people aged 7-10 years, mean age 10.5 ± 3.03, were enrolled in the study. The control group consisted of 50 children at the same age. The body posture was assessed using the photogrammetric method based on the projective moiré pattern [14]. One should also take into account the results of Grabara et al. research, which indicate that the shape of lumbar lordosis is related to age and gender. The authors report that lumbar lordosis decreases with age in male children and adolescents. Their study included a group of 331 girls and 286 boys aged 8 to 16 years. They only qualified children and youth who did not participate in professional sports training but participated in obligatory PE classes. The body posture was assessed using the photogrammetric method. The authors state that girls were characterized by greater lordosis than boys in all ages, except for 10-year-olds. In turn, thoracic kyphosis was significantly higher at the age of 8 and 11 years in boys compared to girls [1]. Also Lichota conducted the study related to the subject, assessing changes in the range of anterior-posterior spinal curvature in children aged 6-7 years. The research showed that the boys' posture was characterized by a greater inclination angle in the upper thoracic and lumbar-sacral segment compared to girls, while the differences concerning the inclination angle of the thoracic-lumbar section were minimal. However, in follow-up after a year, it was found that the inclination of the anterior-posterior curvatures increased significantly, especially in the thoracic-lumbar inclination [39].

In our studies, the analysis of the influence of body mass, height, and BMI on curvature of the spine showed statistically significant relationships in the following parameters: sacral inclination (ALPHA 1), which increased with body weight and BMI in the study group, lumbosacral angle (ALPHA 2), which increased with the body height in the study group, the angle of the middle thoracic Kyphosis TKA DH, which decreased with body weight and height in the study group, and the inclination of the upper thoracic segment (GAMMA C7), which increased with body weight, height, and BMI in the control group.

Grabara et al. analysed the influence of body mass, height, and BMI on the curvature of the spine in children who do not participate in professional sports training and found weak correlations between curvatures in the sagittal plane and somatic parameters such as height, weight, and BMI [1]. Also, Mucha et al. measured somatic features in their research: body weight, height, and BMI as well as the foot length, width, and the surface of the plantar sides of the feet. In order to assess the level of body height and weight, BMI, and the state of the feet arches, the authors compared the results of their own research with those made by Lizis. It was demonstrated that the body height and weight, as well as the BMI index, of a 21-year-old master-class athlete in ju-jitsu are at a level higher than in the peers. The foot length and width were comparable with the peers in the control group. The level of longitudinal and transverse arching was within the normal range, while the plantar foot area differed slightly from one another [24].

Summing up, it can be concluded that the results of our research do not confirm the few reports available in the literature, indicating lack of statistically significant differences between the size of lumbar lordosis and thoracic kyphosis between children practicing karate and the control group. Traditional Karate affects the pelvis tilt leading to posterior tilt, at the same time correlating with somatic parameters such as height, body mass, and BMI in terms of spine curvatures. It is assumed that this may be the result of differences in the time of practicing karate in the above quoted reports, since they cover a time interval of 2 to 10 years of regular participation in training. Therefore, further research is necessary considering the division into groups depending on the time of practicing this martial art.

## 5. Conclusions

Traditional karate affects the change of pelvis tilt to posterior tilt. Among children practicing traditional karate, body height, body mass, and BMI correlate with parameters characterizing spine curvatures in the thoracic and lumbar-sacral segments. The size of the lumbar lordosis and the thoracic kyphosis in people training traditional karate is comparable to that of a group not practicing sport. Frequent incidence of reduced pelvic tilt in traditional karate practitioners requires introduction of exercises activating anterior tilt in a training session.

## Figures and Tables

**Table 1:** Characteristics of anthropometric parameters depending on the group.

Variables	Group	N	*x*± SD	Median	Min	Max	Z	p
Body weight [kg]	karate	76	38.67±12.56	36.85	19.90	66.80	-0.226	0.820
Body weight [kg]	controls	76	39.79±14.90	36.05	20.00	93.00		

Body height [m]	karate	76	1.41±0.16	1.38	1.12	1.78	-2.134	0,031*∗*
Body height [m]	controls	76	1.47±0.17	1.47	1.00	1.86		

BMI	karate	76	18.77±3.06	18.24	14.51	26.37	2.027	0,041*∗*
BMI	controls	76	18.00±3.15	17.38	11.76	27.08		

Z - result of Mann–Whitney *U* test; p - level of probability; N - number of observations; *x* - arithmetic mean; Me - median; SD - standard deviation; *∗* significance at p<0.05

**Table 2:** Comparison of spine parameters in the sagittal plane depending on the group.

Variables	Group	n	*x* ± SD	Me	Min	Max	Z/**t**	p
ALPHA 1	karate	76	8.79±7.94	8.00	0.00	32.00	-4.46	<0.0001*∗∗∗*
ALPHA 1	controls	76	13.47±6.53	12.00	1.00	26.00		

ALPHA 2	karate	76	14.71±6.52	14.00	2.00	32.00	-0.65	0.518
ALPHA 2	controls	76	15.34±5.68	15.50	6.00	29.00		

BETA Th/L	karate	76	14.26±5.50	14.00	4.00	28.00	2.28	0,023*∗*
BETA Th/L	controls	76	11.97±5.62	12.00	0.00	24.00		

GAMMA C7	karate	76	22.37±5.32	22.00	12.00	38.00	-1.84	0.066
GAMMA C7	controls	76	24.67±8.28	24.00	8.00	40.00		

DELTA Th3	karate	76	17.26±6.42	18.00	0.00	30.00	-0.66	0.508
DELTA Th3	controls	76	18.36±6.89	18.00	4.00	34.00		

LLA	karate	76	28.97±7.70	29.50	12.00	50.00	**1.30**	**0.192**
LLA	controls	76	27.32±7.90	28.00	10.00	52.00		

TKA	karate	76	36.63±8.44	36.00	24.00	58.00	-0.34	0.733
TKA	controls	76	36.64±11.10	36.00	10.00	60.00		

TKA DH	karate	76	31.53±8.70	32.00	12.00	54.00	**0.83**	**0.402**
TKA DH	controls	76	30.33±8.89	31.00	10.00	54.00		

ALPHA - sacral inclination; ALPHA 2 – angle of the medial point of the sacrum on the line connecting the so-called Venus dimples; BETA TH /L - intervertebral space Th12- L1; GAMMA C7 - intervertebral space C7-Th1, DELTA Th3 - intervertebral space Th3-Th4 - inclinometer in the plateau of kyphosis; LLA - To obtain the lumbar lordosis on angle – ALPHA 2 + BETA Th / L; TKA - the thoracic kyphosis angle BETA Th12 / L1 + GAMMA C7; TKA DH - Dowager's hump angle GAMMA C7 + DELTA Th3/Th4); n - number of observations; *x* - arithmetic mean; Me- median; SD - standard deviation; **t** - result of Student's t-test for independent variables; Z - result of Mann–Whitney *U* test; p - level of probability; *∗* significance at p<0.05; *∗∗* significance at p<0.001; *∗∗∗*significance at p <0.0001

**Table 3:** Characteristics of changes in the inclination of the sacrum depending on the group.

Sacrum ALPHA 1	Karate Group	Controls Group	Total
Normal (15°-30°)			
FLATTENING	62	43	105

Column %	81.58	56.58	

Total %	40.79	28.29	69.08

NORMAL	12	33	45

Column %	15.79	43.42	

Total %	7.89	21.71	29.61

INCREASED	2	0	2

Column %	2.63	0.00	

Total %	1.32	0.00	1.32

Total	76	76	152

Chi square	15.238		

P	0,0005*∗∗*		

Cramér's V	0.316		

p - level of probability; *∗* significance at p<0.05; *∗∗* significance at p<0.001; *∗∗∗* significance at p<0.0001

**Table 4:** Characteristics of changes in the size of the lumbar lordosis depending on the group.

Lumbar Lordosis Angle (LLA)	Karate Group	Control Group	Total
Normal (30°-40°)			
FLATTENING	38	50	88

Column %	50.00	65.79	

Total %	25.00	32.89	57.89

NORMAL	34	24	58

Column %	44.74	31.58	

Total %	22.37	15.79	38.16

INCREASED	4	2	6

Column %	5.26	2.63	

Total %	2.63	1.32	3.95

Total	76	76	152

Chi square	4.027		

P	0.133		

Cramér's V	0.162		

P - level of probability

**Table 5:** Characteristics of changes in the size of the thoracic kyphosis depending on the group.

Thoracic Kyphosis Angle (TKA)	Karate Group	Control Group	Total
Normal (30°-40°)			
FLATTENING	16	16	32

Column %	21.05	21.05	

Total %	10.53	10.53	21.05

NORMAL	38	32	70

Column %	50.00	42.11	

Total %	25.00	21.05	46.05

INCREASED	22	28	50

Column %	28.95	36.84	

Total %	14.47	18.42	32.09

Total	76	76	152

Chi square	1.234		

P	0.539		

Cramér's V	0.093		

P - level of probability

**Table 6:** Relationship between parameters characterizing the shape of the spine in the sagittal plane with body height and mass, BMI depending on the group.

Spearman's rank-order correlation				
A pair of variables	Karate group (N=76)		Control group (N=76)	
	R Spearman	p	R Spearman	p
Body weight & ALPHA 1	0.31	0,007*∗*	-0.02	0.897

Body weight & ALPHA 2	0.19	0.102	0.02	0.840

Body weight & BETA Th/L	-0.12	0.313	-0.04	0.747

Body weight & GAMMA C7	0.16	0.164	0.31	0,007*∗*

Body weight & DELTA Th3	-0.17	0.147	0.01	0.919

Body weight & LLA	0.13	0.271	-0.08	0.503

Body weight & KTA	0.04	0.763	0.21	0.065

Body weight & KTA DH	-0.24	0,038*∗*	-0.02	0.840

Body height & ALPHA 1	0.09	0.460	0.1	0.389

Body weight & ALPHA 2	0.28	0,015*∗*	0.06	0.597

Body height & BETA Th/L	-0.16	0.179	-0.06	0.635

Body height & GAMMA C7	0.08	0.480	0.23	0,041*∗*

Body height & DELTA Th3	-0.09	0.433	-0.01	0.936

Body height & LLA	0.2	0.080	-0.03	0.806

Body height & KTA	-0.05	0.677	0.14	0.221

Body height & KTA DH	-0.23	0.047	-0.05	0.641

BMI & ALPHA 1	0.46	0,000*∗∗∗*	-0.11	0.338

BMI & ALPHA 2	-0.03	0.775	0.03	0.827

BMI & BETA Th/L	-0.09	0.452	-0.04	0.703

BMI & GAMMA C7	0.2	0.086	0.28	0,014*∗*

BMI & DELTA Th3	-0.2	0.084	0.02	0.891

BMI & LLA	-0.09	0.426	-0.11	0.336

BMI &TKA	0.09	0.441	0.20	0.087

BMI &TKA DH	-0.21	0.068	-0.02	0.865

ALPHA - sacral inclination; ALPHA 2 - angle of the medial point of the sacrum on the line connecting the so-called Venus dimples; BETA TH /L - intervertebral space Th12- L1; GAMMA C7 - intervertebral space C7-Th1, DELTA Th3 - intervertebral space Th3-Th4 - inclinometer in the plateau of kyphosis; LLA - To obtain the lumbar lordosis on angle – ALPHA 2 + BETA Th / L; TKA - the thoracic kyphosis angle BETA Th12 / L1 + GAMMA C7; TKA DH - Dowager's hump angle GAMMA C7 + DELTA Th3/Th4); N - number of observations; p - level of probability; *∗* significance at p<0.05; *∗∗* significance at p<0.001; *∗∗∗* significance at p<0.0001;

## Data Availability

The data used to support the findings of this study are available from the corresponding author upon request.

## Glossary

Gravitational inclinometer: An instrument for measuring inclination of surface with respect to vertical line
MVPA: (Moderate-to-Vigorous Physical Activity) indicator of the level of cumulative motor activity performed with the intensity leading to respiratory increase, considered sufficient for children and adolescents, e.g., sport activities, running from school to school, and PE lessons.

## Additional Points

*Highlights*. Karate does not affect the size of lumbar and thoracic kyphosis but affects the inclination of the sacral bone, correlating simultaneously with somatic parameters.
